# Psychiatric Impact of Organized and Ritual Child Sexual Abuse: Cross-Sectional Findings from Individuals Who Report Being Victimized

**DOI:** 10.3390/ijerph15112417

**Published:** 2018-10-31

**Authors:** Johanna Schröder, Susanne Nick, Hertha Richter-Appelt, Peer Briken

**Affiliations:** Institute for Sex Research and Forensic Psychiatry, University Medical Center Hamburg-Eppendorf, 20246 Hamburg, Germany; s.nick@uke.de (S.N.); hrichter@uke.de (H.R.-A.); briken@uke.de (P.B.)

**Keywords:** child sexual abuse, family violence, organized abuse, ritual abuse, post-traumatic stress disorder, dissociative identity disorder, somatoform dissociation, child maltreatment

## Abstract

Organized and ritual child sexual abuse (ORA) is often rooted in the child’s own family. Empirical evidence on possible associations between ORA and trauma-related symptoms in those who report this kind of extreme and prolonged violence is rare. The aim of our study was to explore socio-demographic and clinical characteristics of the individuals reporting ORA experiences, and to investigate protective as well as promotive factors in the link between ORA and trauma-related symptom severity. Within the framework of a project of the *Independent Inquiry into Child Sexual Abuse* in Germany, we recruited 165 adults who identified themselves as ORA victims via abuse- and trauma-specific networks and mailing lists, and they completed an anonymous online survey. We used variance analyses to examine correlations between several variables in the ORA context and PTSD symptoms (PCL-5) as well as somatoform dissociation (SDQ-5). Results revealed a high psychic strain combined with an adverse health care situation in individuals who report experiences with ORA. Ideological strategies used by perpetrators as well as Dissociative Identity Disorders experienced by those affected are associated with more severe symptoms (η^2^_p_ = 0.11; η^2^_p_ = 0.15), while an exit out of the ORA structures is associated with milder symptoms (η^2^_p_ = 0.11). Efforts are needed to improve health care services for individuals who experience severe and complex psychiatric disorders due to ORA in their childhood.

## 1. Introduction

Child sexual abuse (CSA), once thought to be rare, is nowadays accepted as a frequent reality that occurs across a range of cultures and socioeconomic backgrounds worldwide and encompasses many types of sexually abusive acts towards children, including sexual assault, incest, the production and use of child pornography, as well as commercial sexual exploitation [1]. Sexual child abuse involving a network of perpetrators acting repeatedly and jointly on multiple victims is defined as ‘organized abuse’ [2]. Organized abuse that follows a (pseudo-) ideological strategy (e.g., symbols or group activities with religious, magical, or supernatural connotations) in order to frighten and intimidate the children or to force the victims to participate whilst simultaneously accomplishing the perpetrators’ exculpation is referred to as ‘ritual abuse’ [3,4]. Salter further describes ritual abuse as ideological framing in organized CSA contexts, functioning as strategical practices through which abusive groups indoctrinate the victims into a violently misogynistic worldview in order to control them [4]. In other words, ritual abuse occurs when a religious, political, or spiritual authority uses its position of power and the sovereignty to interpret the respective belief system to manipulate and dominate its followers. Since the 1980s, evidence of organized and ritual abuse (ORA) has been consolidated due to studies documenting psychological harm amongst children and adults disclosing such experiences [5]. At the same time, critics often denied the existence of ritual abuse (e.g., in the context of satanic groups or cults), and dismissed it as the product of false memory phenomena [6]. The described negation of ORA in research, as well as in society, made it difficult to collect the type of empirical data, which would increase confidence in the knowledge of practitioners who recurrently report on their clients’ ORA experiences, including prolonged and repeated interpersonal violence [7] being perpetrated by strangers or as part of abusive networks across families or communities [8]. Studies published in the 90s, that were based on the investigation of alleged cases of ritual abuse, found corroborating evidence for this subtype of organized abuse in some of the analyzed cases [9,10]. Several years later, high profile convictions in cases where ritual abuse may have taken place have been described in a book presenting case analyses in the context of CSA [11]. More recently, doubtful voices were being raised, criticizing previous researchers [12,13], saying that they had misapplied the term ‘ritual abuse’, misstated the facts of many cases, used evidence selectively, and exaggerated the number of false convictions [14,15]. In turn, opposition voices were raised, disapproving of the methodological approaches of the critics and highlighting the complexity of evaluating evidence produced across journalism, law, and science [16,17]. One article, reviewing the described controversy in the context of ORA, came to the conclusion that there are no easy answers to the reasons for the rise and fall of ritual abuse allegations [18]. That is also the reason why research has lacked evidence on the prevalence rates of ORA cases until now.

Clients who identify as victims of ORA present themselves in a range of health and welfare contexts, and report complex clinical pictures of severe trauma-related and dissociative disorders [19]. Health care professionals, who support clients reporting ORA experiences, observe clinical syndromes that go beyond clinical criteria of post-traumatic stress disorders (PTSD), which are primarily interpersonal disturbances, negative self-concepts, and affect dysregulation [20,21]. Individuals with complex PTSD tend to show higher dissociation scores than those with PTSD, and dissociation scores are further related to fear of relationships and withdrawal from shame-evoking situations [22]. Dissociative disorders, characterized by disruptions and/or discontinuities during the normal processes of consciousness, memory, identity, emotion, perception, body representation, motor control, and behavior [23], have been frequently attributed to severe trauma experienced during early childhood [24]. Dissociative experiences, ranging from mild detachment from current surroundings to severe detachment and identity fragmentation, allow for psychological protection through detachment when fight/flight responses are impossible [25]. Evidence suggests a link between accumulated exposure to various types of trauma (e.g., sexual, physical, and emotional abuse) and severity of dissociation symptoms [26]. Females of a German sample with either dissociative identity disorder (DID), which is the most severe syndrome of this spectrum, or a dissociative disorder not otherwise specified (DDNOS), which comprises clinical pictures that do not meet full but similar criteria, suffered from five comorbid diagnoses on average, whereas most of them had a clinically-diagnosed PTSD comorbidity [27,28,29]. Somatoform dissociation (SD) is another clinical picture, which is often related to traumatic experiences, especially child sexual abuse [30,31] or exposure to cumulative trauma and bodily threat [32]. Nijenhuis and colleagues introduced the concept of SD, referring to dissociative symptoms, which phenomenologically involve the body, and comprise a reduction up to a complete loss of sensory perception and/or motor control, as well as involuntary perception of sensory (e.g., prickling), motor (e.g., tremors) and/or pain symptoms [33]. The appearance of such symptoms, after prolonged and repeated trauma, can be explained by the concept of the defense cascade: existential threats first prompt excessive physiological arousal (to prepare the organisms for fight/flight responses). Upon lack of escape options, this arousal turns into immobility due to activation and inhibition of particular functional components as a last way out when faced with an inescapable threat [34,35,36]. Those recurring response patterns in the limbic system are tied in with the original trauma, and are reactivated in contexts of high arousal, even if the danger has already passed [35].

Previous research suggests a plethora of promotive and protective factors in childhood that influence the development of or resilience to psychic strain, consisting of individual (e.g., psychological) and environmental (e.g., parenting and peer) factors [37]. Exposure to CSA leads to psychopathology and psychiatric morbidity [38]. A meta-analysis of the published research on the effects of CSA revealed an average weighted effect of *d* = 0.40 for PTSD, whereby gender, socioeconomic status, type of abuse, age when abused, relationship to perpetrator, and number of abuse incidents, were not found to influence this effect [39]. The authors therefore rather adopt a multifaceted traumatization model, discarding a specific sexual abuse syndrome in the context of CSA. However, a more recent study found that children who were sexually abused by relatives develop more severe PTSD symptoms [40]. Further, a recent study revealed that PTSD correlates with somatization in sexually-abused children, whereby this effect was shown to be moderated by the type of abuse [41]. Some studies revealed evidence on associations between ORA experiences and trauma-related psychopathology in individuals who reported this particularly severe and prolonged form of CSA [19,42,43].

Since no empirical evidence on detailed characteristics of individuals reporting ORA experiences and the relations between different ORA specific variables and trauma-related symptom severity in this group has yet been published, shedding light on this topic would enrich the existing literature substantially. Therefore, the first aim of the current study was to examine socio-demographic and clinical sample characteristics, variables concerning reported ORA experiences, as well as use of health care in individuals who identify themselves as ORA victims. The second aim was to investigate the correlations between common conditions in ORA and trauma-related symptom severity, operationalized by PTSD and SD. According to the previously-described study that suggests there is greater harm when children are sexually abused by relatives [40], we assumed that an involvement of the birth family, which is described to be common in ORA [8], may be associated with increased trauma-related symptom severity. If a structure like the family, which should have a supportive function, turns out to be destructive, we deem an increased insecurity, and thus, conclude that psychopathology is likely. Second, we explored if DID, which are described to be common in individuals who report ORA experiences [44], could be associated with the severity of trauma-related symptoms. It can be assumed both, that dissociative personality states serve as a psychic resilience factor for trauma-related symptom severity, as well as that dissociative personality states reduce the efficiency of trauma treatment in psychotherapy. This is why we kept expectations about the direction of the assumed effect open. Third, we explored if ideological strategies used by perpetrators could affect trauma-related symptom severity. Since the use of (pseudo-)ideological strategies by the perpetrators is described as having the purpose of frightening, intimidating, and controlling victims [4], it can be expected that such abuse contexts promote the development of trauma-related symptoms more strongly than abuse contexts without organized abuse, and without ritual elements. On the other hand, it could be possible that such belief systems serve to give the violence, which the victims reported to have experienced, a meaning, which could also have protective effects on the development of trauma-related symptoms. Therefore, we kept expectations about the direction of the assumed effect open. Fourth, we expected that an exit out of the perpetrator circles would be associated with decreased trauma-related symptoms. The expectation that these variables have a potent association is grounded on the consideration that mind control or programming techniques are described as playing a significant part in keeping victims in control, so that they will not leave the group or expose the perpetrators criminal practices to outsiders [44]. We assumed that the strength to deal with such psychological pressure successfully would correlate with mental resilience. Furthermore, we assumed that the absence of the influence of the perpetrator group as a very potent stress factor must be associated with symptom amelioration. Fifth, we assumed that psychotherapeutic treatment would be associated with decreased trauma-related symptoms. Despite the fact that treating individuals with ORA experiences, especially those with DID, is described as being challenging, it is also described to be helpful when therapists follow certain therapeutic principles (e.g., ideological neutrality) [45].

## 2. Materials and Methods

### 2.1. Study Design

This study presents cross-sectional analyses of data collected in a project studying the healthcare situation of individuals who identify themselves as ORA victims in Germany. This project was funded by the Independent Inquiry into Child Sexual Abuse (UKASK; *Unabhängige Kommission zur Aufarbeitung Sexuellen Kindesmissbrauchs*) in Germany. This group of professionals was appointed by the German Federal Ministry of Family Affairs, Senior Citizens, Women, and Youth (BMFSFJ: *Bundesministerium für Familie*, *Senioren*, *Frauen und Jugend*). The funding body had no role in the design of the study, data collection, analysis, or interpretation of the data. The trial was approved by the ethics committee of the Federal Chamber of Psychotherapists Hamburg (ethical approval code: 03/2017-PTK-HH), and conducted in compliance with the Declaration of Helsinki [46]. The data presented in this article are derived from one anonymous online-survey.

### 2.2. Participants

Participants who identified themselves as ORA victims were recruited online via multiple settings, including the mailing lists and homepages of several CSA/ORA/trauma networks as well as nine chambers of psychotherapists in Germany. We used standardized sheets with detailed information in order to disseminate the aims and conditions of the study. Inclusion criteria were adulthood (age ≥ 18), self-rated psychic stability, and electronic confirmed consent to participate in an anonymous online-survey. The statement that served as key criterion to establish ORA experiences read as follows: “I belong to the target group of the survey, i.e., I have had experiences with organized/ritual violence”, which was defined as “experiences of child sexual abuse (beginning at an age ≤ 16 years) committed by a network of perpetrators acting repeatedly and jointly on multiple victims, whereby certain (religious or political) ideologies or belief systems may have played a role”. The exclusion criterion was a reporting of a repeated participation and/or a reporting of not being a person of interest for the study, that is, an ORA victim by our explicit definition (as stated above), which we examined again in one item at the end of the survey.

### 2.3. Measures

We developed one questionnaire with at least 44 or at most 90 items, mostly consisting of independent single- or multiple-choice questions, as well as open questions in terms of text boxes. The amount of items presented to one participant depended on the experiences that he/she reported. The questions addressed the following topics: socio-demographic variables, awareness and withdrawal, counseling, psychotherapy, psychiatric hospital stays, general support, ORA experiences and their personal consequences, expert assessments, posttraumatic and dissociative symptoms, resources, and validation of the online-survey.

The *PTSD-Checklist for DSM-5* (PCL-5) [47] is a self-reporting measure for PTSD that has been shown to have strong psychometric properties [48], and has been validated for its use in online surveys [49]. Internal consistency of the German PCL-5 version is high, with α = 0.95 for the total scale [50]. Each of the 20 items asks participants how much they were bothered by a given problem related to an event (in this case, ORA), in the past month. The items are grouped into the four clusters of the PTSD section in the DSM-5 [51]. It is used for various purposes, such as screening individuals for PTSD, making a provisional PTSD diagnosis, and monitoring symptom change during or after treatment. The rating is Likert-scaled from 0 (not at all) to 4 (extremely); therefore, the summed up symptom severity score ranges from 0 to 80. A provisional PTSD diagnosis can be made by treating each item rated as 2 (moderate) or higher as a symptom endorsed, then following the DSM-5 diagnostic rule, which requires at least one item related to the dimension ‘intrusions’, one item related to the dimension ‘avoidance’, two items related to the dimension ‘negative cognitions and emotions’, and two items related to the dimension ‘hyperarousal’. Preliminary validation work recommends a PCL-5 cut-point of 33 to propose a PTSD diagnosis [47,50].

The *Somatoform Dissociation Questionnaire-5* (SDQ-5) [52] is a screening instrument for somatoform dissociation with sound psychometric quality and usability for research purposes [53]. The items include insensitivity to pain (analgesia), sensations of disappearing body parts (kinaesthetic anaesthesia), retraction in the visual field (visual anaesthesia), difficulties in speaking (motor disturbance), and pain while urinating, which can be related to sexual trauma [52]. These items are supplied with a 5-point Likert scale, ranging from 1 (applies to me not at all) to 5 (applies to me extremely), with a total score ranging from 5 to 25. The respondent is also asked to indicate whether a physician had connected the symptom or experience with a physical disease. As suggested by the authors, we did not adjust the item scores when physical diseases were indicated, as such indications seemed not to be accurate in the scale development sample [54]. The author suggests optimal sensitivity and specificity at a cut-off value ≥ 8 points [25].

### 2.4. Data Analysis

Statistical analyses were performed in SPSS 21 (IBM^®^, Armonk, NY, USA, 2012, [55]). A multivariate analysis of variance (MANOVA) was calculated in order to examine associations between composite trauma-related symptom severity (operationalized by PCL-5 and SDQ-5) and five variables: ‘birth family involved’ (yes = 1/no = 0), ‘ideological strategies used by perpetrators’ (yes = 1/no = 0), ‘Dissociative Identity Disorder’ (yes = 1/no = 0), ‘exit out of ORA structures’ (yes = 1/no = 0), and ‘psychotherapy’ (yes = 1/no = 0). We chose MANOVA over one-way ANOVA since the dependent variables were correlated (*r* = 0.50, *p* ≤ 0.000). The correlation structure between dependent variables provides MANOVA with beneficial capabilities: enhanced statistical power, assessable patterns between the dependent variables, and a restriction of the joint error rate [56]. The data were screened for violation of statistical conditions for MANOVA prior to the analyses: Examination of scatterplots and histograms was done in order to ascertain linearity and normality. An examination of case-wise diagnostics (Cook’s distance, leverage values), and studentized as well as non-standardized standard deviations of residuals was performed in order to detect cases with undue influence on the model. The value of the *Durbin-Watson* statistic was examined in order to ascertain independence of errors. Multicollinearity was ruled out if correlations between all variables were below 0.7, tolerance was greater than 0.1, and the VIF was smaller than 10. The MANOVA prerequisite of multivariate normality was assumed to be met due to univariate normality. Since the *p* value for the Box Test was 0.554, there was no evidence that the covariance matrices were significantly unequal. Due to mandatory replies to the respective items in the online-assessment, there were no missing data in the continuous dependent variables of PCL-5 and SDQ-5. Hypotheses were tested two-tailed and with an α-level of 0.05. Univariate tests within the multivariate model were performed Bonferroni corrected, in order to eliminate alpha value inflation. Effect sizes are reported as partial eta squared: η^2^_p_ ≈ 0.01 small, η^2^_p_ ≈ 0.06 moderate, η^2^_p_ ≈ 0.14 large effect [57]. The multivariate effect size was calculated based on Roy’s largest root.

## 3. Results

### 3.1. Data Validity

In order to examine the validity of the online self-report data, we conducted several plausibility checks: First, we tested if there were any untrustworthy open answers in the text fields regarding form and content (i.e., text passages without content or answers not fitting the posed questions). Second, we verified that the chronological order of numerical data made sense (e.g., the number of ‘onset of abuse’ had to be lower than the one for ‘awareness of abuse’, and this number had to be lower than the one of ‘age’, respectively), which was given in every case. In both delineated aspects, the ratings of the authors were completely concordant. Third, we checked reliability of the two psychometrical instruments: an α-value of 0.92 in the PCL-5 suggests excellent internal consistency, and a value of 0.65 in the SDQ-5 suggests moderate internal consistency. In conclusion, we can attest that the reports of the participants appear highly accurate.

### 3.2. Sample Characteristics

We reached 167 individuals for eligibility assessment, of whom 165 met the aforementioned inclusion- and exclusion-criteria. Of the two excluded individuals, one case stated that he/she did not fit the target group and the other case stated an onset of sexual abuse at an age higher than 16 years, which did not fit our initial definition of CSA. Table 1 presents socio-demographic sample characteristics. Most of the participants were female, 40 years of age on average, highly educated, in no partnership, retired or incapacitated, had no children, and had grown up in small- or medium sized towns. The most reported sexual orientation was heterosexuality; however, asexuality, homosexuality or bisexuality reached two-digit numbers each.

Table 2 presents sample characteristics referring to ORA experiences reported by the self-identified victims. The reported beginning of ORA ranged from birth to an age of 16 years. On average, participants reported becoming aware of ORA experiences at an age of 29 years. Awareness mostly occurred through sudden flashbacks (76.4%) or situations that triggered memories (66.1%). It took them, on average, 24 years from the beginning until the disclosure of those experiences, where almost all of them confided their abuse experiences to someone. Most participants reported that their birth family was involved in the perpetrator circles, and most of them involved (pseudo-)ideologies (i.e., ritual abuse) or dissociative split strategies (i.e., psychological manipulation or mind control). The self-identified victims reported ORA in the context of child prostitution (66.7%), production of child pornography (64.8%), and/or violent pornography (46.7%). About half of the participants reported that the perpetrators used Satanic ideologies (48.5%), whereas a smaller percentage reported the use of religious (19.4%), fascist (12.1%), racist (12.1%), and/or radical right-wing (8.5%) ideologies. Most participants (74.8%) reported that they were forced to use violence against other individuals themselves. Three quarters of them managed to exit the ORA structures.

Table 3 presents clinical sample characteristics, which refer to mental health care and psychic distress. Almost all of the self-identified ORA victims have had several experiences with outpatient psychotherapy, of which more were based on psychodynamic (69.6%) than on cognitive-behavioral approaches (51.3%). Many of the participants reported that their psychotherapists used trauma-therapeutic interventions (65.8%), and about half of them reported that they encountered one or more psychotherapist(s) with experience in the field of ORA (54.4%). Three quarters reported experiences with inpatient treatment, and a little more than half of them used counselling (see Table 3). Almost all of the self-identified ORA victims reported dissociative personality states, three quarters show an indication for PTSD operationalized by the PCL-5, and even more of them show an indication for SD operationalized by the SDQ-5 (see Table 3).

Table 4 presents life-time and point prevalence rates of psychiatric diagnoses, which the participants reported having received by mental health care professionals (psychiatrist or psychotherapist), and which were, importantly, not clinically validated within the framework of our study. The most frequently-mentioned diagnoses were depressive disorders, (C)PTSD, DID, PTSD and eating disorders. Most of the participants with at least one current psychiatric diagnosis (*n* = 160) rated their current medical outcome as appropriate (84.4%), some as partly appropriate (13.8%), and only a minority as inappropriate (1.9%). A majority of 69.1% participants stated that they, according to their own opinion, were at least once in their lifetime diagnosed wrongly by mental health care professionals. The most frequent diagnoses that were reported to be given by mistake as stated by this subgroup (*n* = 114) in open comments were ‘Emotionally Unstable Personality Disorders’ or ‘Borderline Personality Disorders’ (43.9%), ‘Schizophrenia’ (21.1%), and ‘Psychotic Disorders’ (22.0%). In the same subgroup, 28.9% of the participants reported that the incorrect medical outcome resulted in an application of inadequate psychotherapeutic methods, 25.4% reported a lack of effective psychotherapeutic methods, and 36.0% reported inadequate psychopharmacological treatment.

### 3.3. Associations between ORA-Related Variables and with Trauma-Related Symptoms

Table 5 shows that there were three statistically-significant correlations between the variables that were deemed as factors which may be associated with trauma-related symptoms. First, there was a positive moderate correlation between the variables ‘family involved’ and ‘ideological strategies used’. Second, there was a positive small to moderate correlation between the variables ‘Dissociative Identity Disorder’ and ‘ideological strategies used’. Third, there was a positive moderate to large correlation between the variables ‘psychotherapy’ and ‘Dissociative Identity Disorder’.

Table 6 shows that statistical outcomes revealed a significant large effect of the variable ‘Dissociative Identity Disorder’, increasing PCL-5 (η^2^_p_ = 0.06) as well as SDQ-5 (η^2^_p_ = 0.14) symptoms, resulting in a large overall effect (η^2^_p_ = 0.15). Furthermore, they revealed significant moderate to large effects of the variable ‘exit out of ORA structures’, decreasing PCL5 (η^2^_p_ = 0.08), as well as SDQ-5 (η^2^_p_ = 0.07) symptoms, resulting in an overall moderate to large effect (η^2^_p_ = 0.11). The variable ‘ideological strategies used by perpetrators’ was associated with higher SDQ-5 (η^2^_p_ = 0.11) symptoms, whereas this variable showed no statistical influence on PCL-5 symptoms, still resulting in an overall moderate to large multivariate effect (η^2^_p_ = 0.11). The variable ‘psychotherapy’ revealed a non-significant statistical trend with a small to moderate effect size towards PCL-5 symptom amelioration and no statistically significant association with the SDQ-5. The variable ‘family involved in ORA structures’ revealed effects being far from significant in both PCL-5 and SDQ-5.

## 4. Discussion

The aim of the current study was to explore sample characteristics of German individuals who identified themselves as ORA victims, by means of an anonymous online survey. Another aim was to examine the associations between ORA practices and the use of health care with trauma-related symptom severity (PTSD and SD). The most obvious finding to emerge from the descriptive statistics is that the current sample has very special socio-demographic and psychopathological characteristics. First, it consists mostly of female participants. One possible explanation for this might be that males fall victim to ORA structures a lot less frequently than females, which is in line with sex ratios reported by previous studies on child sexual abuse [58], and also with an Australian sample of self-identified ORA victims (*n* = 21) with 76.2% females [4]. An additional explanation might be that females appear to be seeking help from mental health care services more frequently than males [59], and to take part in online-surveys more often than males [60].

Compared to a large, nationwide, random-sampled study in Germany [61], the current sample shows a larger ratio of non-heterosexual orientation (69.7% vs. 4.1%), that is a higher ratio of exclusively or mainly homosexuality (12.7% vs. 1.9%), a higher ratio of bisexuality (10.9% vs. 1.8%), and a higher ratio of asexuality (26.7% vs. 0.3%). This finding broadly supports the work of other studies in this area linking maltreatment in childhood with same-sex sexuality in adulthood [62], assuming, for example, that sexual abuse of girls by a male perpetrator causes female victims to be aversive to sexual relationships with men [63]. Likewise, the assumption can be made that CSA affects sexual interest or asexuality in adulthood, taking into account that there is as yet no empirical evidence. This is an important issue for future research. Another comparison to results of Matthiesen and colleagues [61] reveals that self-identified ORA victims show a smaller partnership ratio (42.4% vs. 78.0%), which may be explained by the indicated sexual aversion as well. Further, this is in line with results of a group of researchers, who found significant associations between reported CSA and increased sexual problems, as well as disruptions of intimate relationships by difficulties with trust [64]. All these findings seem to be consistent with the result that the parenthood ratio in the current sample of self-identified ORA victims is low (31.5%), compared to age-matched German females (about 79%) according to the BMFSFJ [65].

Participants report repressed memory recovery with a large latency, possibly indicating a pronounced appearance of dissociative amnesia, which serves the purpose of allowing a child to maintain attachment to a person on whom they are dependent for survival in spite of severe traumatic experiences [66]. Fragmented and disorganized trauma memories are known to get more coherent, organized, and detailed as treatment progresses [67]. In this regard, it is relevant that participants in the current study reported an extensive use of mental health care (counseling, inpatient and outpatient treatment, number of professional supporters). This may be caused by the symptom severity, as well as by our recruitment strategy: we focused on networks, mailing lists and homepages that were ORA-specialized, and mental stability was an inclusion criterion. Using this strategy, we may have reached relatively viable individuals, who were likely to use the health care system (e.g., psychotherapy), and who may also be more likely to report that they have managed their exit out of ORA structures. This may reduce the generalizability of our results onto the population of ORA victims still being abused or receiving no support from health care services.

Most participants reported being professionally diagnosed with dissociative disorders (F44 in ICD-10) and experiencing dissociative personality states (indicating DID or DDNOS). This result is also corroborated by the outcomes of the psychometric psychopathological measures on trauma-related symptom severity in the current study, which show indications of PTSD (operationalized by PCL-5) and clinically-relevant SD (operationalized by SDQ-5) in most of the participants. The high psychic strain of the current sample, reflected by psychometric measures as well as reported prevalence rates of psychiatric diagnoses, is in line with the evidence-based impact of child sexual abuse on (C)PTSD and dissociative disorders [68] and with reports of professionals who treat ORA victims [19,69]. The reported prevalence rates are further in line with previous research that demonstrated a strong relationship between childhood trauma and the development of borderline personality disorders [70], eating disorders [71], PTSD and depression [39]. Under the previous assumption that the current sample of self-identified ORA victims may be relatively viable, the psychic strain is alarming. The reported prevalence rates of wrong or inaccurate diagnoses of psychiatric disorders by health care professionals in the current sample are led by Emotionally Unstable or Borderline Personality Disorders (BPD) and Schizophrenia. Taking into account that the design of the study did not allow the researchers to validate them clinically, this could be attributed to the clinical picture of DID, which includes dissociative symptoms like “feelings of unreality” [72] and hallucination symptoms like “voices coming from inside” [72]. It may be possible that health care professionals without experience in treating trauma more frequently attribute dissociative and hallucination symptoms to classic psychiatric diagnoses like schizophrenia or BPD than to controversial and/or neglected disorders like DID [73]. A possible high rate of inaccurate psychiatric diagnoses by health care professionals may also result in an application of inadequate psychotherapeutic methods, a lack of effective psychotherapeutic methods, or inadequate psychopharmacological treatment, and may lead to a poor health care situation despite the previously-proposed good integration into health care structures. The possible inaccuracy of the participants’ appraisal regarding incorrect diagnoses is associated with a limited validity, and suggests a more precise elaboration in future research, for example, by examining this topic in a sample of psychotherapists, who are experienced in diagnosing and treating individuals with ORA experiences.

Correlation analyses revealed an association between family involvement in ORA structures and ideological strategies used by perpetrators, suggesting that family members play a larger role in organized-ritual compared to organized perpetrator groups. This is in line with observations from case studies on victims reporting ritual abuse [74]. Second, there was an association between ideological strategies used by perpetrators and DID, suggesting that perpetrator circles or cults using (pseudo-)ideologies result in higher rates of psychoform dissociation in their group members/victims. The correlation between having used psychotherapy and DID can be explained by the fact that individuals in therapy get diagnoses, while individuals without therapy experiences do not.

Results of the MANOVA indicated that DID reported by the participants is associated with increased PTSD and SD symptoms. This is in line with [22], who revealed empirical evidence suggesting that a clinical level of dissociation correlates with higher CPTSD symptoms. The authors see dissociation as an organizing construct within CPTSD. The results of the current study further revealed that a reported exit out of the ORA structures decreases PTSD and SD symptom severity. It makes sense that trauma-related symptoms ameliorate when the damaging influences of the perpetrators have disappeared. The results further suggest that the use of (pseudo-)ideological strategies by the perpetrators, that is, according to our definition, ritual abuse, increases trauma-related symptom severity. Since the use of (pseudo-)ideological strategies by the perpetrators is deemed to have the exact purpose of frightening, intimidating, and controlling their victims [4], it is understandable that this affects SD symptoms more strongly than organized abuse without ritual elements. A possible explanation, given the statistical trend towards PTSD symptom deterioration, is that higher statistical power would be essential in order to detect the small to moderate effect shown in the current study. Analyses further failed to detect a statistically-significant influence of psychotherapeutic treatment on trauma-related symptoms. Ahead of discussing possible reasons, it is important to mention that there is some scientific evidence for the existence of effective therapeutic methods for the treatment of CPTSD and dissociative disorders [23,75]. The statistically non-significant effect in the current sample may be related to the severity of the trauma sequelae, to high rates of psychiatric morbidity, but also to inaccurate diagnoses made by professionals, followed by inadequate psychotherapeutic as well as psychopharmacological treatment. Further, conventional psychotherapy mostly focusses on cognitive processing and prolonged exposure when treating trauma, which should be supplemented with a phasic approach that addresses the manifold dimensions of symptoms by also focusing on dissociation [23]. Further, CSA in the ORA context has been reported to be associated with factors resulting in severe long-term harm, such as multiple perpetrators, more frequent incidents of abuse, and longer periods of abuse [2], which may impede psychotherapeutic processes to a great extent [7]. Another possible explanation is that this binary variable revealed rather low variance (over 90% of the participants reported psychotherapy experiences), causing high type II error probability (failing to detect an effect), which is a statistical limitation. Besides, our definition of trauma-related symptoms does not cover the whole clinical picture, since a high ratio of the current sample reports more complex disorders like DDNOS and DID. Therefore, future studies should examine the influence of psychotherapy not only on PTSD and SD, but also on psychoform dissociation symptoms. The result that involvement of the birth family in the perpetrator circles is not associated with trauma-related symptom severity in self-identified ORA victims gives rise to questions, since the impact of sexual abuse upon victims is deemed to be related to factors like the child’s familial and community environment, as well as the relationship between perpetrator and victim [76]. Since previous research suggests that individuals who were sexually abused by relatives resulted in worse outcomes [40], future studies should therefore examine factors that possibly moderate or mediate the effect of family involvement on trauma-related symptom severity in ORA victims, e.g., developing psychoform dissociation symptoms like DID.

One general limitation that has to be considered when interpreting our results is that data were collected in an anonymous online-study. In order to accomplish absolute anonymity of participants for security reasons, we did not collect IP-addresses, which makes it impossible to prevent multiple log-ins by using cookies. There is no proof for the validity of the collected data (e.g., for participants’ fitting the inclusion criteria or for no multiple log-ins), but detailed plausibility checks during the process of data cleaning evoked confidence in valid instead of random answers. Furthermore, it is possible that the participants’ beliefs about prior childhood trauma are attributable to adult maladjustment and false memories [77]. Then again, we observed a huge group of participants who provided very congruent data on variables in the ORA context and severe clinical symptoms, which will not solve the current false memory debate, and researchers should therefore investigate them independently from that discussion. Another limitation that should be considered when interpreting the presented results is that possibly confounding variables (e.g., socioeconomic status or substance use), possible biases (e.g., through retrospective self-reports), and reverse causality restrict drawing firm conclusions about the direction of the described effects. For example, it is theoretically possible that individuals with more severe psychiatric problems have an increased risk of falling victim to abusive perpetrator circles, and further, that individuals with more severe trauma-symptoms have limited access to psychotherapy, and therefore, fail to leave the perpetrator circles. Also, it is yet unclear, and therefore, should be investigated if the appearance of ORA revealed in the current study can be generalized to other countries, considering for example the networking structures in organized abuse, the content of ideologies in ritual abuse, or the health care support that individuals with ORA experiences receive. Another statistically-limiting factor was the answering option “I don’t know”: on the one hand it may have enhanced the validity of the responses, but, on the other, it may have reduced the statistical test power. In future studies, is therefore necessary to explore more promotive and protective factors that may be associated with trauma-related symptom severity in individuals reporting ORA experiences. It would further be interesting to realize longitudinal study designs in order to be able to detect development over time.

## 5. Conclusions

ORA, defined as organized child sexual abuse where a (pseudo-)ideological (i.e., ritual) content serves as legitimization for violence, is a complex and polarizing issue in mental health care contexts as well as in research. At present, the uncovering and reprocessing of ORA is a problem that remains to be solved in Germany, as well as internationally. Given the paucity of research in this field, we believe that this study contributes to closing this evidence gap, as it presents empirical data on reported practices of ORA and its impact on trauma-related symptom severity in self-identified victims, in which reported ideological/ritual strategies by the perpetrators and an exit out of the ORA structures play a major role. A key policy priority should therefore be to intensify efforts on the understanding of ORA-related structures, as well as the complex clinical presentation of those affected. Services like information websites and exit programs should be developed by experts in the field in order to contribute to generating appropriate treatment services for this group of clients. Mental health professionals and centers specialized in the treatment of severely traumatized clients with CPTSD and dissociative disorders would contribute to a better support of clients who report such trauma histories. The therapeutic process of detachment from perpetrator networks is intense, and supporters of individuals who experience ORA face special difficulties, like, for example, dissociative personality states. Therefore, it is essential to ensure that constant professional supervision is provided to them by professionals who understand the spectrum of possibilities and pitfalls in the treatment.

## Figures and Tables

**Table 1:** Socio-demographic sample characteristics of self-identified ORA victims (*n* = 165).

Variables	Statistics	
Age in years		
M (SD), Mdn	40.0 (10.3)	39
Sex		
Female (*n*, %)	158	95.8
Male (*n*, %)	4	2.4
Other (*n*, %)	3	1.8
Sexual orientation		
Exclusively/mainly heterosexual (*n*, %)	50	30.3
Bisexual (*n*, %)	18	10.9
Exclusively/mainly homosexual (*n*, %)	21	12.7
Asexual/no sexual interests (*n*, %)	44	26.7
Unsure (*n*, %)	32	19.4
Partnership status		
No partnership (*n*, %)	95	57.6
Partnership (*n*, %)	70	42.4
Parentship		
Children (*n*, %)	52	31.5
No children (*n*, %)	113	68.5
Graduation		
Lower secondary school (*n*, %)	8	4.8
Middle secondary school (*n*, %)	61	37.0
Higher secondary school (*n*, %)	95	57.6
Not yet completed, other or no degree (*n*, %)	1	0.6
Employment status		
Full or part time employment (*n*, %)	32	19.4
Marginal employment (*n*, %)	10	6.1
No employment (*n*, %)	14	8.5
In professional education (*n*, %)	9	5.5
Retired or incapacitated (*n*, %)	100	60.6
Size of former hometown; number of inhabitants		
Major city; ≥100.000 (*n*, %)	56	33.9
Small- or medium-sized town; ≥5.000 (*n*, %)	65	39.4
Village/countryside (*n*, %)	44	26.7

Notes: M—mean; SD—standard deviation; Mdn—median; ORA—organized and ritual abuse.

**Table 2:** Experiences of self-identified victims in the ORA context (*n* = 165).

Variables		Statistics	
Onset of ORA (age in years)			
*n* = 109	M (SD), Mdn.	3.0 (0.3)	2.0
Awareness of ORA experiences (age in years)			
*n* = 146	M (SD), Mdn.	28.5 (12.4)	26.5
Disclosure			
*n* = 160	Yes (*n*, %)	156	97.5
	No (*n*, %)	4	2.5
Time from beginning of ORA to diclosure (in years)			
*n* = 156	M (SD), Mdn.	24.2 (13.5)	23.0
Birth family involved			
*n* = 165	Yes (*n*, %)	132	80.0
	No (*n*, %)	33	20.0
Ideological strategies used by perpetrators			
*n* = 122	Yes (*n*, %)	107	87.7
	No (*n*, %)	15	12.3
Dissociative split strategies used by perpetrators			
*n* = 151	Yes (*n*, %)	96	63.6
	No (*n*, %)	55	36.4
Exit out of ORA structures			
*n* = 125	Yes (*n*, %)	94	75.2
	No (*n*, %)	31	24.8

Notes: M—mean; SD—standard deviation; Mdn.—median. Missing data = answer option “I don’t know”.

**Table 3:** Clinical sample characteristics of self-identified ORA victims (*n* = 165).

Variables		Statistics	
Use of counseling			
*n* = 165	Yes (*n*, %)	106	64.2
	No (*n*, %)	59	35.8
Use of outpatient psychotherapy			
*n* = 165	Yes (*n*, %)	158	95.8
	No (*n*, %)	7	4.2
Use of inpatient treatment			
*n* = 165	Yes (*n*, %)	122	73.9
	No (*n*, %)	43	26.1
Indication of PTSD (PCL-5)			
*n* = 165	Yes (*n*, %)	125	75.8
	No (*n*, %)	40	24.7
	Total score (M, SD)	51.44 (16.25)	
Indication of somatoform dissociation (SDQ-5)			
*n* = 165	Yes (*n*, %)	147	89.1
	No (*n*, %)	18	10.9
	Total score (M, SD)	14.36 (4.44)	

Notes: M—mean; SD—standard deviation; ORA—organized and ritual sexual child abuse; PTSD—posttraumatic stress disorder; PCL-5—PTBS-Checklist-5; SDQ-5—Somatoform Dissociation Questionnaire-5; missing data = answer option “I don’t know”.

**Table 4:** Prevalence rates of psychiatric disorders diagnosed by health care professionals, according to self-reports by self-identified ORA victims (*n* = 165).

Psychiatric Disorders	Professionally Diagnosed in Lifetime (%, *n*)	Professionally Diagnosed Currently (%, *n*)
Depressive Disorder	87.3 (144)	51.5 (85)
Complex Posttraumatic Stress Disorder	84.8 (140)	53.9 (89)
Dissociative Identity Disorder	83.6 (138)	78.8 (130)
Posttraumatic Stress Disorder	73.9 (122)	23.6 (39)
Eating Disorder	62.4 (103)	24.2 (40)
Dissociative Disorder	51.5 (85)	18.2 (30)
Generalized Anxiety Disorder	42.4 (70)	10.9 (18)
Borderline Personality Disorder	42.4 (70)	4.8 (8)
Emotionally Unstable Personality Disorder	40.0 (66)	3.6 (6)
Panic Disorder and/or Agoraphobia	32.1 (53)	11.5 (19)
Social Anxiety Disorder	25.5 (42)	10.3 (17)
Substance Abuse and/or Addiction	24.8 (41)	4.2 (7)
Schizophrenia	24.8 (41)	0.6 (1)
Enduring Personality Change after Catastrophic Experience	20.6 (34)	7.3 (12)
Psychotic Disorder	19.4 (32)	0.6 (1)
Other Personality Disorder	18.8 (31)	3.6 (6)
Delusional Disorder	17.6 (29)	0.6 (1)
Obsessive-Compulsive Disorder	17.0 (28)	3.0 (5)
Dissociative Disorder Not Otherwise Specified	12.7 (21)	3.0 (5)

Notes: ORA—organized and ritual sexual child abuse. Indications are ordered by prevalence. The following indications with prevalence rates below 10% in both categories were left unspecified: Bipolar Affective Disorder, Specific Phobia, Attention Deficit Hyperactivity Disorder, Somatoform Disorder, Autism Spectrum Disorder. The reported diagnoses were not clinically validated within the framework of this study.

**Table 5:** Correlations between ORA-related and clinical variables in 165 self-identified victims.

	1	2	3	4	5
1. Family involved in ORA structures	-				
2. Ideological strategies used by perpetrators	*n* = 122, *r* = 0.32, *p* ≤ 0.001 *	-			
3. Dissociative Identity Disorder	*N* = 165, *r* = 0.04, *p* = 0.636	*n* = 122, *r* = 0.25, *p* = 0.005 *	-		
4. Exit out of ORA structures	*n* = 125, *r* = −0.10, *p* = 0.258	*n* = 95, *r* = −0.13, *p* = 0.223	*n* = 125, *r* = −0.05, *p* = 0.620	-	
5. Psychotherapy	*N* = 165, *r* = −0.03, *p* = 0.701	*n* = 122, *r* = 0.05, *p* = 0.596	*N* = 165, *r* = 0.41, *p* ≤ 0.001 *	*n* = 125, *r* = 0.11, *p* = 0.239	-

Notes: ORA—organized and ritual child sexual abuse. Missing data = answer option “I don’t know”. Statistical significance at 0.05 level = *.

**Table 6:** Univariate and multivariate analyses of variance, exploring the association between ORA-related and clinical variables with trauma-related symptoms in 165 self-identified victims.

	PCL-5 (ANOVA)	SDQ-5 (ANOVA)	MANOVA
Family involved in ORA structures	*F*(1,89) = 0.312; *p* = 0.578; η^2^_p_ = 0.00; β = −0.06	*F*(1,89) = 0.302; *p* = 0.584; η^2^_p_ = 0.00; β = −0.05	Λ_Roy_ = 0.005; *F*(2,88) = 0.221; *p* = 0.802; η^2^_p_ = 0.01
Ideological strategies used by perpetrators	*F*(1,89) = 1.718; *p* = 0.193; η^2^_p_ = 0.02; β = 0.14	*F*(1,89) = 11.433; *p* = 0.001 *; η^2^_p_ = 0.11; β = 0.32	Λ_Roy_ = 0.128; *F*(2,88) = 5.654; *p* = 0.005 *; η^2^_p_ = 0.11
Dissociative Identity Disorder	*F*(1,89) = 6.107; *p* = 0.015 *; η^2^_p_ = 0.06; β = 0.26	*F*(1,89) = 14.715; *p* ≤ 0.001 *; η^2^_p_ = 0.14; β = 0.37	Λ_Roy_ = 0.179; *F*(2,88) = 7.898; *p* = 0.001*; η^2^_p_ = 0.15
Exit out of ORA structures	*F*(1,89) = 8.000; *p* = 0.006 *; η^2^_p_ = 0.08; β = −0.27	*F*(1,89) = 6.767; *p* = 0.011 *; η^2^_p_ = 0.07; β = −0.23	Λ_Roy_ = 0.121; *F*(2,88) = 5.330; *p* = 0.007 *; η^2^_p_ = 0.11
Psychotherapy	*F*(1,89) = 3.291; *p* = 0.073; η^2^_p_ = 0.04 β = −0.19	*F*(1,89) = 0.325; *p* = 0.570; η^2^_p_ = 0.00; β = −0.05	Λ_Roy_ = 0.037; *F*(2,88) = 1.633; *p* = 0.201; η^2^_p_ = 0.04

Notes: PCL-5—PTSD Checklist-5; SDQ-5—Somatoform Dissociation Questionnaire-5; ORA—organized and ritual child sexual abuse; Λ_Roy_—Roy’s largest root; Effect sizes: η^2^_p_ ≈ 0.01 small, η^2^_p_ ≈ 0.06 moderate, η^2^_p_ ≈ 0.14 large. Univariate tests within the multivariate model were performed with Bonferroni-correction. Standardized regression weights were obtained from linear regressions. Statistical significance at 0.05 level = *. A sensitivity analysis revealed that, given α = 0.05 and β = 0.80, an effect size of η^2^_p_ = 0.05 was required to reveal statistically significant results.
